# Tumeur à cellules géantes du fémur proximal traitée par arthroplastie totale de hanche

**DOI:** 10.11604/pamj.2016.23.93.9046

**Published:** 2016-03-15

**Authors:** Younes Mhammdi, Mohamed Kharmaz

**Affiliations:** 1 Service de Traumatologie Orthopédie, Hopital Ibn Sina, CHU Rabat, Maroc

**Keywords:** Tumeur à cellules géantes, fémur Proximal, PTH, Giant cell tumor, proximal femur, THP

## Abstract

The authors report the case of a giant cell tumor grade 1 of the proximal femur, diagnosed in a young patient of 18 years. The diagnosis was suspected on a standard x-ray complemented by MRI, it was confirmed on histological examination after biopsy. The tumor was a metaphyseal-epiphyseal location above the level of the femoral cut on preoperative planning. The management had combined two surgical techniques, with a marginal resection of the femoral head and neck, and femoral curettage-filling of the metaphyseal region with implantation of a total cemented hip prosthesis. After 2 years of follow up, the result is very satisfactory with good functional recovery and no signs of recurrence.

## Image en médecine

Les auteurs rapportent le cas d'une tumeur à cellules géantes grade 1 de l'extrémité supérieure du fémur, diagnostiquée chez une jeune patiente de 18 ans. Le diagnostic a été suspecté sur une imagerie standard complétée par une IRM est a été confirmé sur une étude histologique après biopsie. La tumeur avait une localisation épiphyso-métaphysaire dépassant le niveau de coupe fémoral sur la planification préopératoire. La prise en charge avait combiné deux techniques chirurgicales, avec une résection marginale de la tête et du col fémoral, et un curetage comblement de la région métaphysaire fémorale avec mise en place d'une PTH cimentée. Le résultat est très satisfaisant avec une bonne récupération fonctionnelle sans aucun signe de récidive après un recul de 2 ans.

**Figure 1:**
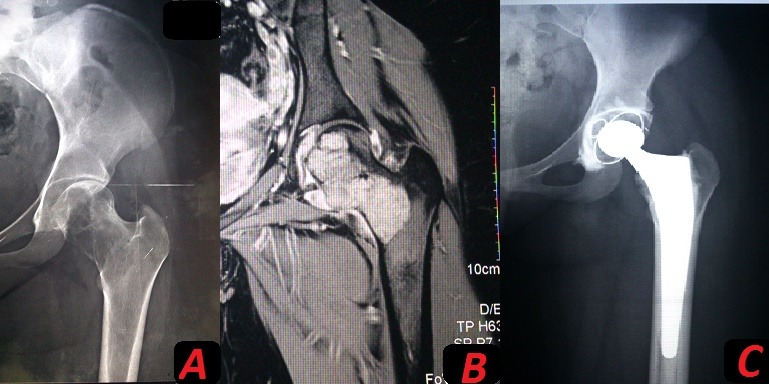
(A): aspect typique d'une TCG sur l'imagerie standard localisée au fémur proximal; (B): aspect IRM de la même tumeur du fémur proximal; (C): radio de contrôle montrant une prothèse totale de hanche cimentée en place après résection puis curetage de la tumeur

